# Ethyl [(benzyl­aza­nium­yl)(2-hy­droxy­phen­yl)meth­yl]phospho­nate

**DOI:** 10.1107/S1600536811026444

**Published:** 2011-07-09

**Authors:** Xiangdong Zhang, Rui Zhang, Chunhua Ge, Xiaoyan Zhang

**Affiliations:** a College of Chemistry, Liaoning University, Shenyang, Liaoning 110036, People’s Republic of China

## Abstract

The title compound, C_16_H_20_NO_4_P, crystallizes as a zwitterion. In the mol­ecule, the two aromatic rings form a dihedral angle of 55.2 (1)°. In the crystal, inter­molecular N—H⋯O and O—H⋯O hydrogen bonds link the mol­ecules into columns propagating in [010].

## Related literature

For related structures, see: Zhang *et al.* (2005 ▶, 2007 ▶).
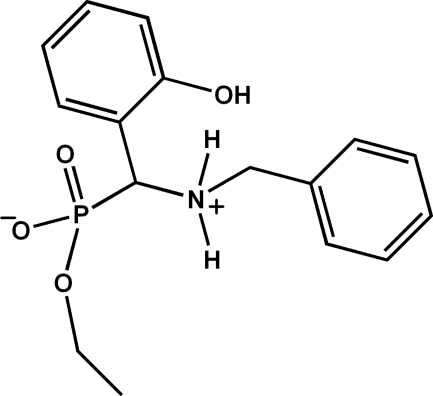


## Experimental

### 

#### Crystal data

C_16_H_20_NO_4_P
*M* *_r_* = 321.30
Monoclinic, 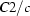

*a* = 28.069 (3) Å
*b* = 6.0927 (7) Å
*c* = 22.333 (3) Å
β = 124.464 (2)°
*V* = 3149.0 (6) Å^3^
*Z* = 8
Mo *K*α radiation
μ = 0.19 mm^−1^
*T* = 296 K
0.28 × 0.25 × 0.19 mm

#### Data collection

Bruker SMART CCD area-detector diffractometer
Absorption correction: multi-scan (*SADABS*; Bruker, 2001 ▶) *T*_min_ = 0.955, *T*_max_ = 0.969
8453 measured reflections
3104 independent reflections
1918 reflections with *I* > 2σ(*I*)
*R*_int_ = 0.057

#### Refinement

*R*[*F* ^2^ > 2σ(*F* ^2^)] = 0.049
*wR*(*F* ^2^) = 0.121
*S* = 1.00
3104 reflections
209 parameters
H atoms treated by a mixture of independent and constrained refinement
Δρ_max_ = 0.30 e Å^−3^
Δρ_min_ = −0.28 e Å^−3^

### 

Data collection: *SMART* (Bruker, 2001 ▶); cell refinement: *SAINT* (Bruker, 2001 ▶); data reduction: *SAINT*; program(s) used to solve structure: *SHELXS97* (Sheldrick, 2008 ▶); program(s) used to refine structure: *SHELXL97* (Sheldrick, 2008 ▶); molecular graphics: *SHELXTL* (Sheldrick, 2008 ▶); software used to prepare material for publication: *SHELXL97*, *PLATON* (Spek, 2009 ▶) and *WinGX* (Farrugia, 1999 ▶).

## Supplementary Material

Crystal structure: contains datablock(s) I, global. DOI: 10.1107/S1600536811026444/cv5124sup1.cif

Structure factors: contains datablock(s) I. DOI: 10.1107/S1600536811026444/cv5124Isup2.hkl

Supplementary material file. DOI: 10.1107/S1600536811026444/cv5124Isup3.cml

Additional supplementary materials:  crystallographic information; 3D view; checkCIF report

## Figures and Tables

**Table 1:** Hydrogen-bond geometry (Å, °)

*D*—H⋯*A*	*D*—H	H⋯*A*	*D*⋯*A*	*D*—H⋯*A*
N1—H1*NA*⋯O1^i^	0.97 (3)	1.77 (3)	2.738 (3)	179 (2)
N1—H1*NB*⋯O4	0.86 (3)	2.50 (3)	2.982 (3)	116 (2)
N1—H1*NB*⋯O2^ii^	0.86 (3)	2.08 (3)	2.915 (3)	164 (2)
O4—H4*A*⋯O1	0.82	1.94	2.738 (3)	164
O4—H4*A*⋯O2^ii^	0.82	2.58	2.925 (3)	107

Symmetry codes: (i) 
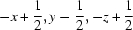
; (ii) 
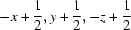
.

**Fig. 1.**
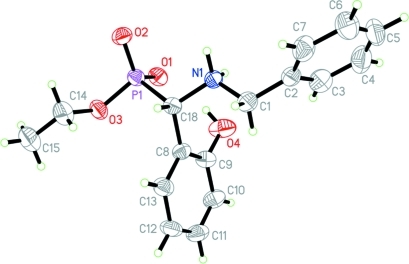
The molecular structure of the title compound, with displacement ellipsoids drawn at the 50% probability level.

## supplementary crystallographic information

### Comment

As a continuation of our structural study of (2-hydroxyphenyl)methylphosphonate derivatives (Zhang *et al.*, 2005; 2007), we present here the crystal structure of the title compound, (I), which crystallizes as a zwitterion.

In the title molecule (Fig. 1), two aromatic rings form a dihedral angle of 55.2 (1)°. In the crystal structure, intermolecular N—H···O and O—H···O hydrogen bonds (Table 1) link molecules into columns propagated in [010].

### Experimental

The title compound was synthesized following the reported method (Zhang *et al.*, 2005). Diethyl phosphonate (0.02 mol) was dissolved in 80 ml of ethanol. The solution was added dropwise to a mixture of salicylaldehyde (0.02 mol) and benzylamine (0.02 mol) in 30 ml e thanol which was refluxed for 4 h. The resulting solution was refluxed until solid appeared. The product was filtered and washed with ethanol. Yield 43%.

### Refinement

H atoms attached to C atoms were positioned geometrically and refined using a riding model, with C*sp*^3^—H = 0.96–0.98 Å or C*sp*^2^—H = 0.93 Å and with *U*_iso_(H) = 1.2*U*_eq_(C). H atom attached to O atom was O—H = 0.82 Å and with *U*_iso_(H)=1.5*U*_eq_(O). H atoms attached to N atom were located by difference Fourier synthesis with the range 0.86–0.97 Å and refined isotropically.

### Crystal data

**Table:**

C_16_H_20_NO_4_P	*F*(000) = 1360
*M**_r_* = 321.30	*D*_x_ = 1.355 Mg m^−^^3^
Monoclinic, *C*2/*c*	Mo *K*α radiation, λ = 0.71073 Å
Hall symbol: -C 2yc	Cell parameters from 187 reflections
*a* = 28.069 (3) Å	θ = 2.6–22.5°
*b* = 6.0927 (7) Å	µ = 0.19 mm^−^^1^
*c* = 22.333 (3) Å	*T* = 296 K
β = 124.464 (2)°	Block, colourless
*V* = 3149.0 (6) Å^3^	0.28 × 0.25 × 0.19 mm
*Z* = 8	

### Data collection

**Table:**

Bruker SMART CCD area-detector diffractometer	3104 independent reflections
Radiation source: fine-focus sealed tube	1918 reflections with *I* > 2σ(*I*)
graphite	*R*_int_ = 0.057
φ and ω scans	θ_max_ = 26.0°, θ_min_ = 1.8°
Absorption correction: multi-scan (*SADABS*; Bruker, 2001)	*h* = −34→33
*T*_min_ = 0.955, *T*_max_ = 0.969	*k* = −7→7
8453 measured reflections	*l* = −19→27

### Refinement

**Table:**

Refinement on *F*^2^	Primary atom site location: structure-invariant direct methods
Least-squares matrix: full	Secondary atom site location: difference Fourier map
*R*[*F*^2^ > 2σ(*F*^2^)] = 0.049	Hydrogen site location: inferred from neighbouring sites
*wR*(*F*^2^) = 0.121	H atoms treated by a mixture of independent and constrained refinement
*S* = 1.00	*w* = 1/[σ^2^(*F*_o_^2^) + (0.0504*P*)^2^] where *P* = (*F*_o_^2^ + 2*F*_c_^2^)/3
3104 reflections	(Δ/σ)_max_ = 0.001
209 parameters	Δρ_max_ = 0.30 e Å^−^^3^
0 restraints	Δρ_min_ = −0.28 e Å^−^^3^

### Special details

**Table:**

Geometry. All e.s.d.'s (except the e.s.d. in the dihedral angle between two l.s. planes) are estimated using the full covariance matrix. The cell e.s.d.'s are taken into account individually in the estimation of e.s.d.'s in distances, angles and torsion angles; correlations between e.s.d.'s in cell parameters are only used when they are defined by crystal symmetry. An approximate (isotropic) treatment of cell e.s.d.'s is used for estimating e.s.d.'s involving l.s. planes.
Refinement. Refinement of *F*^2^ against ALL reflections. The weighted *R*-factor *wR* and goodness of fit *S* are based on *F*^2^, conventional *R*-factors *R* are based on *F*, with *F* set to zero for negative *F*^2^. The threshold expression of *F*^2^ > σ(*F*^2^) is used only for calculating *R*-factors(gt) *etc*. and is not relevant to the choice of reflections for refinement. *R*-factors based on *F*^2^ are statistically about twice as large as those based on *F*, and *R*- factors based on ALL data will be even larger.

### Fractional atomic coordinates and isotropic or equivalent isotropic displacement parameters (Å^2^)

**Table:**

	*x*	*y*	*z*	*U*_iso_*/*U*_eq_	
P1	0.17738 (3)	0.87761 (11)	0.22830 (4)	0.0315 (2)	
O3	0.11541 (7)	0.8149 (3)	0.20942 (10)	0.0399 (5)	
N1	0.23047 (10)	0.8251 (4)	0.15689 (12)	0.0303 (5)	
O2	0.22470 (8)	0.7684 (3)	0.29547 (9)	0.0393 (5)	
C18	0.17490 (11)	0.7697 (4)	0.14969 (13)	0.0301 (6)	
H18	0.1735	0.6094	0.1520	0.036*	
C8	0.12149 (11)	0.8387 (4)	0.07710 (13)	0.0315 (6)	
O1	0.17840 (8)	1.1221 (3)	0.22305 (10)	0.0376 (5)	
O4	0.15309 (10)	1.2130 (3)	0.08789 (11)	0.0586 (6)	
H4A	0.1652	1.2047	0.1309	0.088*	
C2	0.28397 (12)	0.7998 (5)	0.09779 (14)	0.0378 (7)	
C10	0.06502 (13)	1.1018 (5)	−0.01849 (15)	0.0448 (7)	
H10	0.0610	1.2415	−0.0376	0.054*	
C1	0.23072 (12)	0.7379 (5)	0.09411 (15)	0.0424 (7)	
H1A	0.2277	0.5791	0.0932	0.051*	
H1B	0.1971	0.7936	0.0492	0.051*	
C13	0.07828 (12)	0.6856 (5)	0.03567 (15)	0.0399 (7)	
H13	0.0826	0.5438	0.0535	0.048*	
C9	0.11301 (12)	1.0518 (4)	0.04989 (15)	0.0375 (7)	
C11	0.02300 (13)	0.9456 (5)	−0.05861 (16)	0.0474 (8)	
H11	−0.0095	0.9812	−0.1042	0.057*	
C7	0.32831 (13)	0.6516 (5)	0.12183 (16)	0.0505 (8)	
H7	0.3259	0.5144	0.1382	0.061*	
C12	0.02899 (12)	0.7382 (5)	−0.03144 (16)	0.0451 (8)	
H12	0.0002	0.6339	−0.0578	0.054*	
C5	0.38007 (15)	0.9030 (7)	0.09799 (19)	0.0648 (10)	
H5	0.4122	0.9375	0.0978	0.078*	
C3	0.28884 (14)	1.0027 (5)	0.07376 (17)	0.0511 (8)	
H3A	0.2595	1.1056	0.0572	0.061*	
C14	0.10333 (14)	0.5962 (5)	0.2229 (2)	0.0629 (10)	
H14A	0.1335	0.5506	0.2718	0.075*	
H14B	0.1024	0.4934	0.1890	0.075*	
C15	0.04702 (15)	0.5964 (7)	0.2139 (2)	0.0851 (13)	
H15A	0.0485	0.6956	0.2483	0.128*	
H15B	0.0386	0.4511	0.2220	0.128*	
H15C	0.0173	0.6426	0.1655	0.128*	
C4	0.33690 (16)	1.0534 (6)	0.07413 (19)	0.0635 (10)	
H4	0.3399	1.1903	0.0581	0.076*	
C6	0.37635 (15)	0.7037 (6)	0.12200 (19)	0.0648 (10)	
H6	0.4060	0.6021	0.1386	0.078*	
H1NA	0.2628 (13)	0.754 (4)	0.1997 (16)	0.050 (9)*	
H1NB	0.2378 (11)	0.963 (4)	0.1627 (14)	0.033 (8)*	

### Atomic displacement parameters (Å^2^)

**Table:**

	*U*^11^	*U*^22^	*U*^33^	*U*^12^	*U*^13^	*U*^23^
P1	0.0278 (4)	0.0342 (4)	0.0320 (4)	−0.0001 (3)	0.0167 (3)	−0.0020 (3)
O3	0.0304 (11)	0.0432 (12)	0.0473 (12)	−0.0015 (9)	0.0227 (10)	0.0046 (9)
N1	0.0311 (14)	0.0301 (14)	0.0293 (13)	0.0002 (11)	0.0169 (12)	−0.0005 (11)
O2	0.0338 (11)	0.0488 (12)	0.0306 (10)	0.0068 (9)	0.0155 (9)	0.0055 (9)
C18	0.0285 (15)	0.0256 (14)	0.0321 (15)	0.0010 (11)	0.0147 (13)	0.0016 (11)
C8	0.0306 (15)	0.0350 (16)	0.0265 (14)	0.0032 (12)	0.0147 (12)	−0.0019 (12)
O1	0.0369 (11)	0.0333 (11)	0.0418 (11)	−0.0045 (9)	0.0218 (10)	−0.0078 (9)
O4	0.0591 (15)	0.0372 (12)	0.0452 (13)	−0.0073 (11)	0.0090 (12)	0.0035 (10)
C2	0.0379 (17)	0.0472 (18)	0.0300 (15)	−0.0016 (14)	0.0202 (14)	−0.0071 (13)
C10	0.0451 (19)	0.0437 (18)	0.0351 (16)	0.0101 (15)	0.0164 (15)	0.0067 (14)
C1	0.0429 (18)	0.0532 (19)	0.0337 (16)	−0.0061 (14)	0.0232 (15)	−0.0111 (14)
C13	0.0319 (17)	0.0465 (18)	0.0390 (16)	−0.0002 (14)	0.0187 (14)	−0.0035 (14)
C9	0.0333 (17)	0.0368 (17)	0.0346 (15)	0.0021 (13)	0.0145 (13)	−0.0016 (13)
C11	0.0347 (18)	0.066 (2)	0.0312 (16)	0.0105 (16)	0.0126 (14)	−0.0002 (16)
C7	0.058 (2)	0.055 (2)	0.0491 (19)	0.0096 (17)	0.0372 (18)	0.0018 (16)
C12	0.0272 (17)	0.060 (2)	0.0375 (17)	−0.0078 (14)	0.0117 (14)	−0.0112 (15)
C5	0.054 (2)	0.096 (3)	0.061 (2)	−0.021 (2)	0.043 (2)	−0.016 (2)
C3	0.057 (2)	0.0476 (19)	0.0494 (19)	0.0017 (16)	0.0301 (17)	−0.0012 (16)
C14	0.054 (2)	0.068 (2)	0.064 (2)	−0.0147 (18)	0.0314 (19)	0.0084 (19)
C15	0.046 (2)	0.119 (3)	0.081 (3)	−0.016 (2)	0.031 (2)	0.024 (2)
C4	0.078 (3)	0.068 (2)	0.062 (2)	−0.020 (2)	0.049 (2)	−0.0062 (19)
C6	0.054 (2)	0.087 (3)	0.066 (2)	0.016 (2)	0.042 (2)	−0.001 (2)

### Geometric parameters (Å, °)

**Table:**

P1—O2	1.4841 (18)	C1—H1B	0.9700
P1—O1	1.4961 (18)	C13—C12	1.384 (4)
P1—O3	1.5874 (18)	C13—H13	0.9300
P1—C18	1.839 (3)	C11—C12	1.370 (4)
O3—C14	1.448 (3)	C11—H11	0.9300
N1—C1	1.503 (3)	C7—C6	1.383 (4)
N1—C18	1.513 (3)	C7—H7	0.9300
N1—H1NA	0.97 (3)	C12—H12	0.9300
N1—H1NB	0.86 (3)	C5—C6	1.355 (5)
C18—C8	1.517 (3)	C5—C4	1.364 (5)
C18—H18	0.9800	C5—H5	0.9300
C8—C13	1.388 (3)	C3—C4	1.380 (4)
C8—C9	1.396 (4)	C3—H3A	0.9300
O4—C9	1.367 (3)	C14—C15	1.477 (4)
O4—H4A	0.8200	C14—H14A	0.9700
C2—C7	1.377 (4)	C14—H14B	0.9700
C2—C3	1.384 (4)	C15—H15A	0.9600
C2—C1	1.498 (4)	C15—H15B	0.9600
C10—C11	1.380 (4)	C15—H15C	0.9600
C10—C9	1.382 (4)	C4—H4	0.9300
C10—H10	0.9300	C6—H6	0.9300
C1—H1A	0.9700		
			
O2—P1—O1	118.69 (11)	C8—C13—H13	119.1
O2—P1—O3	112.33 (10)	O4—C9—C10	118.2 (3)
O1—P1—O3	106.60 (10)	O4—C9—C8	121.3 (2)
O2—P1—C18	109.48 (11)	C10—C9—C8	120.4 (3)
O1—P1—C18	105.86 (11)	C12—C11—C10	120.2 (3)
O3—P1—C18	102.51 (11)	C12—C11—H11	119.9
C14—O3—P1	121.11 (18)	C10—C11—H11	119.9
C1—N1—C18	111.8 (2)	C2—C7—C6	121.0 (3)
C1—N1—H1NA	105.4 (16)	C2—C7—H7	119.5
C18—N1—H1NA	109.7 (16)	C6—C7—H7	119.5
C1—N1—H1NB	111.2 (17)	C11—C12—C13	119.3 (3)
C18—N1—H1NB	112.3 (17)	C11—C12—H12	120.3
H1NA—N1—H1NB	106 (2)	C13—C12—H12	120.3
N1—C18—C8	112.8 (2)	C6—C5—C4	120.4 (3)
N1—C18—P1	109.82 (16)	C6—C5—H5	119.8
C8—C18—P1	113.68 (17)	C4—C5—H5	119.8
N1—C18—H18	106.7	C4—C3—C2	120.6 (3)
C8—C18—H18	106.7	C4—C3—H3A	119.7
P1—C18—H18	106.7	C2—C3—H3A	119.7
C13—C8—C9	117.7 (2)	O3—C14—C15	109.2 (3)
C13—C8—C18	119.2 (2)	O3—C14—H14A	109.8
C9—C8—C18	123.0 (2)	C15—C14—H14A	109.8
C9—O4—H4A	109.5	O3—C14—H14B	109.8
C7—C2—C3	118.1 (3)	C15—C14—H14B	109.8
C7—C2—C1	120.6 (3)	H14A—C14—H14B	108.3
C3—C2—C1	121.2 (3)	C14—C15—H15A	109.5
C11—C10—C9	120.4 (3)	C14—C15—H15B	109.5
C11—C10—H10	119.8	H15A—C15—H15B	109.5
C9—C10—H10	119.8	C14—C15—H15C	109.5
C2—C1—N1	113.0 (2)	H15A—C15—H15C	109.5
C2—C1—H1A	109.0	H15B—C15—H15C	109.5
N1—C1—H1A	109.0	C5—C4—C3	120.1 (3)
C2—C1—H1B	109.0	C5—C4—H4	120.0
N1—C1—H1B	109.0	C3—C4—H4	120.0
H1A—C1—H1B	107.8	C5—C6—C7	119.8 (3)
C12—C13—C8	121.8 (3)	C5—C6—H6	120.1
C12—C13—H13	119.1	C7—C6—H6	120.1

### Hydrogen-bond geometry (Å, °)

**Table:**

*D*—H···*A*	*D*—H	H···*A*	*D*···*A*	*D*—H···*A*
N1—H1NA···O1^i^	0.97 (3)	1.77 (3)	2.738 (3)	179 (2)
N1—H1NB···O4	0.86 (3)	2.50 (3)	2.982 (3)	116 (2)
N1—H1NB···O2^ii^	0.86 (3)	2.08 (3)	2.915 (3)	164 (2)
O4—H4A···O1	0.82	1.94	2.738 (3)	164
O4—H4A···O2^ii^	0.82	2.58	2.925 (3)	107

Symmetry codes: (i) −*x*+1/2, *y*−1/2, −*z*+1/2;  (ii) −*x*+1/2, *y*+1/2, −*z*+1/2.
